# Hazardous alcohol consumption is associated with increased levels of B-type natriuretic peptide: evidence from two population-based studies

**DOI:** 10.1007/s10654-013-9808-9

**Published:** 2013-05-05

**Authors:** David A. Leon, Vladimir M. Shkolnikov, Svetlana Borinskaya, Juan-Pablo Casas, Alun Evans, Artyom Gil, Frank Kee, Nikolay Kiryanov, Martin McKee, Mark G. O’Doherty, George B. Ploubidis, Olga Polikina, Maxim Vassiliev, Stefan Blankenberg, Hugh Watkins

**Affiliations:** 1 London School of Hygiene and Tropical Medicine, London, UK; 2 Max Planck Institute for Demographic Research, Rostock, Germany; 3 New Economic School, Moscow, Russia; 4 Vavilov Institute for General Genetics, Moscow, Russia; 5 Genetic Epidemiology Unit, University College London, London, UK; 6 Queen’s University Belfast, Belfast, UK; 7 I.M. Sechenov First Moscow State Medical University, Moscow, Russia; 8 Izhevsk State Medical Academy, Izhevsk, Russia; 9 Research and Applied Science Centre Bilolazgom, Moscow, Russia; 10 Department of Cardiology, University Heart Center, Hamburg, Germany; 11 Radcliffe Department of Medicine, University of Oxford, Oxford, UK

**Keywords:** Alcohol, B-type natriuretic peptide, Russia, Cardiovascular disease

## Abstract

Russia has very high mortality from cardiovascular disease (CVD), with evidence that heavy drinking may play a role. To throw further light on this association we have studied the association of alcohol with predictors of CVD risk including B-type natriuretic peptide (BNP). Levels of BNP increase primarily in response to abnormal cardiac chamber wall stretch which can occur both as a result of atherosclerosis as well as due to other types of damage to the myocardium. No previous population-based studies have investigated the association with alcohol. We analysed cross-sectional data on drinking behaviour in 993 men aged 25–60 years from the Izhevsk Family Study 2 (IFS2), conducted in the Russian city of Izhevsk in 2008–2009. Relative to non-drinkers, men who drank hazardously had an odds ratio (OR) of being in the top 20 % of the BNP distribution of 4.66 (95 % CI 2.13, 10.19) adjusted for age, obesity, waist–hip ratio, and smoking. Further adjustment for class of hypertension resulted in only slight attenuation of the effect, suggesting that this effect was not secondary to the influence of alcohol on blood pressure. In contrast hazardous drinking was associated with markedly raised ApoA1 and HDL cholesterol levels, but had little impact on levels of ApoB and LDL cholesterol. Similar but less pronounced associations were found in the Belfast (UK) component of the PRIME study conducted in 1991. These findings suggest that the association of heavy drinking with increased risk of cardiovascular disease may be partly due to alcohol-induced non-atherosclerotic damage to the myocardium.

## Introduction

B-type natriuretic peptide (BNP) levels are increased primarily in response to abnormal cardiac chamber wall stretch caused by increased haemodynamic load. This is found in heart failure, as well as a result of damage to the ventricle from cardiomyopathy, ischaemia or other causes [1]. BNP levels are positively associated with risk of cardiovascular events, in those initially free from cardiovascular disease (CVD) [2, 3], without clinical evidence of ventricular dysfunction [4], and at levels far below those used in a clinical triage context to identify those with acute heart failure [2, 5].

A considerable amount of work has been done on the association of BNP levels with other cardiovascular risk factors including smoking [6] and obesity [7]. In addition, there is intriguing evidence that small, genetically determined life-long elevated levels of BNP protect against type II diabetes mellitus, but are not associated with classic cardio-metabolic risk factors including BMI, blood pressure and lipid profiles [8]. However, surprisingly, the association of BNP levels with alcohol consumption has not been systematically investigated in population-based studies. The only exception is a published table from the British Regional Heart Study that indicates that ‘heavy drinkers’ had raised NT-pro BNP levels, although this was not commented upon in the text of the paper [9]. Aside from this, the alcohol-BNP association has only been investigated indirectly in a few small studies of patients with alcoholic (and non-alcoholic) cirrhosis [10–12] and oesophageal varices [13] that aimed to identify whether this hormone could be used as a diagnostic/prognostic marker. However, given continuing uncertainty about the apparent cardio-protective effects of moderate drinking, and increasing evidence that heavy drinking raises the risk of ischaemic heart disease [14] and stroke [15, 16] this is a major gap in the literature.

Russia has exceptionally high mortality from circulatory disease, especially among working-age men, which is the single main reason for their very low life expectancy at birth (63 years in 2010). However, it has been repeatedly noted that lipid profiles from Russian population samples do not appear to be particularly pro-atherogenic: surprisingly high HDL and ApoA1 levels, but unexceptional levels of LDL and ApoB have been found in many studies [17, 18]. In contrast control of blood pressure in Russia is poor [19] and smoking rates among men are high [20]. Most recently, there is increasing evidence that in Russia hazardous alcohol consumption is strongly associated with deaths attributed to ischaemic heart disease [21–24]. This could be due to alcohol promoting atherosclerosis or, alternatively, could reflect misclassification of deaths due to alcohol-induced damage to the myocardium causing ventricular impairment and/or arrhythmias [25, 26] as suggested almost two decades ago [17]. Little is known about the mechanisms that may underlie these associations of hazardous alcohol drinking with cardiovascular disease.

Here we report results of a study of working-age men in the Russian city of Izhevsk in which we investigated associations of alcohol consumption with BNP and other predictors of cardiovascular risk including ApoA1 and ApoB. Given the almost complete absence of published data on the association of alcohol with BNP, we have replicated the analysis using the Belfast (UK) component of the PRIME study [27].

## Methods

### Izhevsk family study

The 2008–2009 Izhevsk Family Study 2 (IFS2) was a cross-sectional survey of 1,531 working-age men (25–60 years), 1,068 of whom had a medical examination. Izhevsk is a typical medium-sized Russian city located on the European side of the Urals. The participants were originally recruited (2003–2006) as an age-stratified random sample from the 2002 population register of adult city residents [23].

Having traced the man to his current address in the city, consent was sought to interview him and a proxy informant (usually his wife or mother) living in the same household. For those consenting, a trained interviewer collected information from both a proxy informant and the man in their own home using structured questionnaires in face-to-face interviews.

For the purposes of these analyses we used information provided by the man himself about socio-demographic characteristics, smoking habits and also about the frequency of drinking each type of beverage alcohol (beer, wine and spirits) over the past year, and the amount of each beverage drunk on a usual occasion. This latter information on alcohol consumption was used to estimate the average volume of ethanol consumed per week by each man, based on the assumption that the concentration of ethanol by volume in beer was 4.5 %, wine was 12 % and vodka and other spirits 43 %.

We also developed a measure of hazardous drinking that was based on frequencies of various behaviours related to either pattern of drinking or its consequences over the preceding year. This included frequency of consumption of non-beverage alcohols such as medicinal tinctures as well as frequency of *zapoi* (a period of drunkenness of two or more days characterised by withdrawal from normal social life). Using this information we defined a hazardous drinker as a man having one or more of the following attributes: in the previous year any consumption of non-beverage alcohols, one or more episodes of *zapoi*, or twice weekly or more occurrence of either hangover, excessive drunkenness or going to sleep at night clothed because of being drunk. As in our previous work, we classified men as hazardous drinkers using proxy-reported information on these aberrant, but easily observable behaviours, as these were considered less likely to be underestimated than self-reports [22, 23, 28].

A medical examination was offered to men following the initial interview. Those men taking part in this second stage provided information themselves in response to a doctor-administered questionnaire. This included standard questions on breathlessness and the Rose angina questionnaire. The distribution of men by age, drinking behaviour and educational level was very similar regardless of whether or not they had a medical examination.

The majority (91 %) of the 1,068 examinations conducted took place at a clinic, the remainder taking place in the subject’s home. Hip and waist circumference, height and weight were measured three times using standard protocols and the mean of all three readings used in analyses. Seated arterial blood pressure was measured three times using Omron (705 IT) electronic sphygmomanometers. The mean of the second and third readings were used in the analyses. Non-fasting blood samples were taken. These were placed in cool bags containing ice and transported to a local laboratory where they were spun in a cooled centrifuge and aliquoted within 12 h of venepuncture. Aliquots not used immediately were stored at −80 °C.

Assays of GGT, Hepatitis B and C were conducted in Izhevsk. GGT was measured using the kinetic colorimetric method [29]. Hepatitis tests were carried out using kits from Vector-Best [30]. Lipid and apolipoprotein assays were conducted in Moscow using thawed samples by Lytech, a commercial diagnostics laboratory using an Architect i2000 analyser. BNP was measured in EDTA plasma using a chemiluminescent microparticle immunoassay (CMIA) produced by Abbott for use on the Architect i2000 analyser. The alcohol biomarker, carbohydrate deficient transferrin (CDT), was measured at the Moscow Research and Practical Center on Addictions using the Sebia Capillarys 2 multicapillary analyser [31].

### Belfast PRIME study

The PRIME study is a multi-centre, prospective cohort study of coronary events established in 1991 whose design is described in detail elsewhere [27]. Briefly, the Belfast component of the PRIME study (referred to simply as the PRIME study hereafter) recruited men aged 50–59 years who broadly matched the social class structure of this Northern Irish city.

At baseline, 2,741 men completed self-administered questionnaires at home that included questions on personal and family history of disease, smoking, alcohol consumption and physical activity. Alcohol consumption was evaluated by assessing the different types of beverage consumed (wine, beer, cider and spirits) and their alcohol content (% alcohol by volume) for each day in the previous week, or in a “usual” week if this was atypical. For each day of the week, the numbers of standard servings (defined explicitly in terms of amounts that respondents would recognise) by beverage type and alcoholic strength were recorded. This was converted into a total equivalent consumption of pure ethanol for the entire week.

Height and weight were measured using a standard protocol. Blood pressure was measured at the end of the examination after a 5 min rest in the sitting position. Measurements were performed with an automatic device (Spengler SP9; Spengler, Asnières sur Seine, France). A standard cuff size was used, but a larger cuff was available when necessary. A fasting blood sample was drawn from each participant, and the plasma was prepared with EDTA and used for analysis of lipids. Plasma total cholesterol and triglycerides were measured by enzymatic methods using reagents from Boehringer Ingelheim (Mannheim, Germany). Apolipoproteins A-1 and B were measured by a nephelometric method (Behringwerke; Marburg, Germany). GGT was measured using a Gamma-Glutamyl-Transferase assay produced by Abbott for use on the Architect c8000. As in IFS2, BNP was measured in EDTA plasma using a chemiluminescent microparticle immunoassay (CMIA) produced by Abbott for use on the Architect i2000. LDL levels were estimated using the Friedwald equation in both IFS2 and the PRIME studies.

### Exposure measures

Alcohol drinking was the primary exposure of interest, for which we had three different measures. The exposure available in both studies was self-reported average weekly consumption of alcohol measured in units of 10 ml pure ethanol derived as described above. For IFS2 we used a second type of exposure measure which characterised pattern of drinking based on proxy observations of frequency of dysfunctional behaviours associated with heavy drinking. This measure was constructed independent of information about actual amount drunk, but has been shown to be highly predictive of cardiovascular mortality in an earlier study [22]. The third measure of exposure to alcohol was more objective, being based on two alcohol biomarkers (GGT and CDT) that were both only available in IFS2. From these we derived a continuous normally distributed biomarker variable that combined information from GGT and CDT using a latent variable modeling approach. Our latent variable measurement model was based on a multivariate logit analysis with latent variables [32]. The measurement model was estimated in the Mplus 6.12 software, with the robust maximum likelihood estimator (MLR) [33].

### Outcome measures

The outcome measures were levels of BNP, other biomarker predictors of CVD risk and prevalence of hypertension. All of the biomarkers were dichotomised according to whether or not values fell into the top 20 % (quintile) of the distribution of each study population. This was done in preference to treating these outcomes as continuous variables for two reasons: (1) over 40 % of the BNP levels in IFS2 fell below the limit of detection (10 pg/ml) and using a cut-offs avoided this problem; (2) it allowed the strength of associations of the alcohol variables with the various outcomes to be directly compared without assumptions concerning the normality of their underlying distribution. Hypertension was treated as a binary variable.

### Other covariates

In the regression models we adjusted for a number of other covariates. Age was included in this category as an obvious potential confounder, as was smoking. However, in addition we also adjusted for body mass index and waist–hip ratio. Both of these markers of adiposity have been found to be predictors of CHD risk, but both have in different populations be associated with alcohol consumption. However, as the focus of our analysis was on whether alcohol per se might have a direct impact on cardiovascular risk factors we adjusted for these markers of adiposity, even though in some populations they might lie on the causal pathway between alcohol and CVD risk.

### Statistical methods

Logistic regression was used to estimate the strength of the association of the the alcohol exposure variables with the outcome biomarkers and hypertension. In these models adjustment was made for BMI and waist–hip ratio in categories to avoid having to make assumptions about the linearity of their associations with the outcomes. For the BNP analyses in IFS2 we also checked that similar inferences could be drawn from the results of tobit (censored) regression. The IFS2 and PRIME analyses were conducted using STATA 11 [34].

Ethical approval for IFS2 2 was granted by committees of the Izhevsk State Medical Academy and the London School of Hygiene & Tropical Medicine, and for the Belfast PRIME study by Queens University Belfast. Participants in both studies gave informed consent compliant with the guidelines of the Declaration of Helsinki.

## Results

Table 1 shows the characteristics of the two study populations. Summary measures for IFS2 are presented for those aged 50–60 years, as well as the total (aged 30–60 years), to allow direct comparison with the PRIME population all of whom were in the age range 50–60 years. Median BMI was very similar in the two studies. However, the percentage obese was higher in IFS2, as were the percentages of smokers and hypertensives. The average number of units of alcohol reported to be consumed per week was higher in PRIME than IFS2. However among drinkers the mean percentage of beverage ethanol consumed as spirits was 59 % in IFS2 but only 24 % in PRIME. In addition, total ethanol consumption in IFS2 was underestimated as it was not possible to take into account ethanol from non-beverage sources, consumed by 7 % of men in the previous year. The percentage of abstainers in IFS2 was much lower than in PRIME (14 vs. 23 %) although of these, nearly all were ex-drinkers in IFS2, while in PRIME they were mostly life-long abstainers. The lipid profile in the PRIME study was appreciably more pro-atherogenic than in IFS2, with Belfast men having higher LDL cholesterol, triglycerides, ApoB and ApoB/ApoA1 ratio (driven mainly by much higher ApoB), while Izhevsk men had higher HDL levels.

**Table 1:** Characteristics of IFS2 and PRIME study populations

	N^a^			Median or %		
	IFS2 (30–60 years)	IFS2 (50–60 years)^b^	PRIME (50–60 years)	IFS2 (30–60 years)	IFS2 (50–60 years)^b^	PRIME (50–60 years)
Age (years)	1,068	558	2,741	50	55	54
Waist–hip ratio	1,064	554	2,741	0.93	0.94	0.94
BMI (kg/m^2^)	1,060	551	2,741	25.9	25.8	26.0
Obese (≥30 kg/m^2^)	1,060	551	2,741	18 %	18 %	12 %
Hypertensive (%) (SBP ≥ 140 mmHg/DBP ≥ 90 mmHg)	1,061	553	2,738	59 %	69 %	40 %
Current smoker (%)	1,068	558	2,741	63 %	60 %	32 %
Units/week of beverage ethanol consumed	1,051	551	2,741	8.5	6.7	8.3
Units/week of beverage ethanol consumed (current drinkers)	915	474	2,101	11.1	10.1	15.9
Former drinkers	1,068	558	2,741	12.4 %	13.3 %	2.2 %
Life-long abstainers	1,068	558	2,741	0.8 %	0.5 %	21.1 %
BNP (pg/mL)	986	523	1,806	11.6	15.1	18.3
Total cholesterol (mmol/L)	981	504	2,733	5.32	5.37	5.85
HDL cholesterol (mmol/L)	981	504	2,733	1.34	1.34	1.16
LDL cholesterol (mmol/L)	963	494	2,679	3.20	3.23	3.89
Triglycerides (mmol/L)	963	494	2,734	1.28	1.29	1.50
Apolipoprotein A1 (g/L)	976	499	2,388	1.42	1.40	1.37
Apolipoprotein B (g/L)	977	500	2,389	0.88	0.90	1.14
Apolipoprotein B/Apolipoprotein A1 ratio	976	499	2,387	0.62	0.64	0.84
Gamma glutamyl transferase (GGT) (IU/L)	1,038	537	2,113	29.7	29.1	36.9
% Carbohydrate deficient transferrin (CDT)	1,012	517	–	1.00	1.00	–

^a^For IFS2 subjects are all men who had been interviewed themselves and had attended the medical examination. For both studies variation in numbers reflect missing information (including blood sample not available for analysis)

^b^To facilitate more direct comparison of characteristics of men in PRIME and IFS2, we have included this column that describes the subset of IFS2 men who were in the same age range as the total PRIME population i.e. 50–60 years

The face-validity of the questionnaire-derived data on alcohol drinking was confirmed in that there were strong, graded associations of the alcohol biomarkers with volume and pattern of drinking (Table 5).

In IFS2 those in the top quintile of the BNP distribution had a BNP value of 27 pg/ml or more, the equivalent value for the PRIME study being 34 pg/ml. Table 2 shows the associations of various factors with being in the top quintile of the BNP distribution in each study. Age was positively associated with raised BNP levels in both studies, as was class of hypertension and self-reports of breathlessness when walking on level ground. However, Rose questionnaire reported angina was only associated with raised BNP levels in PRIME, while smoking only showed an association in IFS2. Class of obesity was not related with raised BNP in either study.

**Table 2:** Association of BNP with selected characteristics in IFS2 and PRIME, adjusted for age

	IFS2			PRIME		
	N	OR	(95 % CI)	N	OR	(95 % CI)
Age (years)						
<35	81	0.27	(0.10, 0.69)	–	–	–
35–39	86	0.25	(0.10, 0.65)	–	–	–
40–44	105	0.33	(0.15, 0.73)	–	–	–
45–49	191	0.88	(0.54, 1.42)	–	–	–
50–54	247	1.00	[ref]	1,006	1.00	[ref]
55–60	276	2.12	(1.42, 3.16)	800	1.89	(1.50, 2.39)
*P* value trend			<0.001			–
Smoking						
Never smoker	192	1.00	[ref]	614	1.00	[ref]
Former smoker	179	1.19	(0.67, 2.14)	688	1.00	(0.76, 1.32)
Current smoker	614	1.91	(1.20, 3.06)	504	0.95	(0.71, 1.29)
*P* value trend			0.002			0.796
Obesity class						
Underweight	19	0.38	(0.08, 1.76)	5	–	–
Normal weight	395	1.00	[ref]	661	1.00	[ref]
Over weight	386	0.77	(0.53, 1.10)	922	0.97	(0.75, 1.24)
Obese	142	0.61	(0.36, 1.04)	180	1.07	(0.71, 1.61)
Severely obese	38	0.65	(0.25, 1.67)	38	1.42	(0.67, 3.03)
*P* value trend			0.104			0.620
Hypertension						
Normotensive	407	1.00	[ref]	1,092	1.00	[ref]
Mild hypertension	309	1.00	(0.66, 1.52)	456	1.04	(0.78, 1.38)
Moderate hypertension	163	1.35	(0.85, 2.15)	178	1.76	(1.22, 2.52)
Severe hypertension	103	2.45	(1.48, 4.05)	78	1.70	(1.01, 2.85)
*P* value trend			0.001			0.001
Angina (Rose questionnaire)						
No	893	1.00	[ref]	1,643	1.00	[ref]
Yes	83	1.44	(0.95, 2.19)	163	2.77	(1.97, 3.90)
Stop for breath walking						
No	934	1.00	[ref]	1,188	1.00	[ref]
Yes	44	2.37	(1.25, 4.52)	139	2.47	(1.66, 3.66)

OR—odds ratio of being in the top 20 % of BNP distribution

For IFS2 subjects are all men who had been interviewed themselves and had attended the medical examination. For both studies variation in total numbers reflect missing information

Obesity class BMI ranges = Underweight (≤18.5); Normal weight (≥18.5–24.9); Over weight (≥25–29.9); Obese (≥30–34.9); Severely obese (≥35)

Hypertension = Mild SBP 140–159 or DBP 90–99; Moderate 160–179 or 100–109; Severe 180+ or 110+

Stop for breath walking based on response to question “Do you have to stop for breath when walking at your own pace on level ground?”

Table 3 shows the association of various markers of alcohol consumption with the risk of hypertension and being in the top (study-specific) quintiles of various cardiovascular biomarkers, adjusted for age, obesity and waist–hip ratio. Adjustment for anthropometric variables was undertaken as they may confound associations of drinking behaviour and outcomes. However, this is a conservative strategy, as it will also largely remove the effects of alcohol on the outcomes that are mediated through body habitus.

**Table 3:** Association of predictors of CVD risk with measures of alcohol consumption in IFS2 and PRIME, adjusted for age, class of obesity and waist–hip ratio quintile

	BNP		HDL cholesterol		LDL cholesterol		Triglycerides		ApoA1		ApoB		ApoB/ApoA1		Hypertension	
	OR	(95 % CI)	OR	(95 % CI)	OR	(95 % CI)	OR	(95 % CI)	OR	(95 % CI)	OR	(95 % CI)	OR	(95 % CI)	OR	(95 % CI)
Izhevsk Family Study 2																
Alcohol biomarker quartiles^a^		N = 963		N = 970		N = 952		N = 952		N = 965		N = 966		N = 965		N = 1,011
1 (bottom)	1.00	[ref]	1.00	[ref]	1.00	[ref]	1.00	[ref]	1.00	[ref]	1.00	[ref]	1.00	[ref]	1.00	[ref]
2	1.06	(0.63, 1.80)	2.42	(1.2, 4.88)	1.62	(0.99, 2.63)	1.65	(0.99, 2.76)	2.33	(1.08, 5.01)	1.76	(1.06, 2.91)	1.06	(0.68, 1.67)	1.31	(0.91, 1.91)
3	2.01	(1.22, 3.32)	5.66	(2.87, 11.1)	1.54	(0.94, 2.53)	1.31	(0.78, 2.19)	6.72	(3.29, 13.7)	1.78	(1.08, 2.94)	0.90	(0.57, 1.42)	2.57	(1.74, 3.80)
4 (top)	2.20	(1.36, 3.57)	24.2	(12.8, 45.7)	1.51	(0.91, 2.48)	1.42	(0.83, 2.43)	23.59	(12.0, 46.4)	1.50	(0.89, 2.54)	0.45	(0.26, 0.76)	3.62	(2.43, 5.38)
*P* value trend		<0.001		<0.001		0.158		0.411		<0.001		0.171		0.004		<0.001
Pattern of drinking (proxy report)		N = 851		N = 853		N = 835		N = 835		N = 848		N = 849		N = 848		N = 919
Non-drinker^b^	1.00	[ref]	1.00	[ref]	1.00	[ref]	1.00	[ref]	1.00	[ref]	1.00	[ref]	1.00	[ref]	1.00	[ref]
Non-hazardous	2.35	(1.20, 4.58)	8.18	(2.92, 22.9)	0.74	(0.46, 1.21)	0.83	(0.49, 1.42)	9.67	(2.99, 31.2)	0.73	(0.44, 1.21)	0.34	(0.21, 0.54)	1.78	(1.18, 2.68)
Hazardous	4.51	(2.14, 9.52)	20.16	(6.88, 59.0)	0.53	(0.27, 1.04)	0.64	(0.31, 1.33)	21.6	(6.44, 72.6)	0.55	(0.28, 1.09)	0.31	(0.16, 0.59)	2.59	(1.52, 4.43)
*P* value trend		<0.001		<0.001		0.064		0.234		<0.001		0.084		<0.001		<0.001
*P* value difference in drinkers		0.005		<0.001		0.239		0.353		<0.001		0.282		0.782		0.093
Alcohol units/week (self-report)^c^		N = 920		N = 915		N = 899		N = 899		N = 911		N = 911		N = 911		N = 990
Non-drinker^b^	1.00	[ref]	1.00	[ref]	1.00	[ref]	1.00	[ref]	1.00	[ref]	1.00	[ref]	1.00	[ref]	1.00	[ref]
<5	1.50	(0.74, 3.03)	4.06	(1.16, 14.2)	0.60	(0.34, 1.04)	0.73	(0.40, 1.32)	3.10	(0.87, 11.0)	0.62	(0.35, 1.09)	0.35	(0.21, 0.60)	1.29	(0.82, 2.03)
5–19	2.93	(1.53, 5.58)	15.8	(4.83, 51.9)	0.84	(0.51, 1.38)	1.00	(0.58, 1.72)	12.2	(3.73, 39.6)	0.82	(0.49, 1.36)	0.33	(0.20, 0.54)	2.07	(1.35, 3.16)
20–44	2.64	(1.27, 5.47)	18.0	(5.28, 61.4)	0.57	(0.31, 1.05)	0.74	(0.39, 1.40)	20.2	(6.01, 67.7)	0.53	(0.28, 0.99)	0.26	(0.14, 0.48)	2.74	(1.64, 4.57)
45+	2.82	(1.17, 6.81)	56.5	(15.8, 202.0)	0.47	(0.21, 1.09)	0.31	(0.11, 0.83)	40.9	(11.6,144.0)	0.30	(0.11, 0.78)	0.19	(0.08, 0.48)	2.64	(1.4, 4.98)
*P* value trend		0.001		<0.001		0.134		0.128		<0.001		0.030		<0.001		<0.001
*P* value trend in drinkers		0.040		<0.001		0.517		0.179		<0.001		0.143		0.099		0.001
Belfast PRIME study																
Alcohol units/week (self-report)		N = 2,738		N = 2,733		N = 2,679		N = 2,734		N = 2,388		N = 2,389		N = 2,387		N = 1,806
Non-drinker^b^	1.00	[ref]	1.00	[ref]	1.00	[ref]	1.00	[ref]	1.00	[ref]	1.00	[ref]	1.00	[ref]	1.00	[ref]
<5	1.32	(1.04, 1.69)	1.50	(1.00, 2.27)	1.01	(0.75, 1.34)	1.21	(0.89, 1.64)	1.51	(1.00, 2.28)	1.31	(0.94, 1.82)	1.03	(0.77, 1.38)	0.97	(0.68, 1.39)
5–19	1.21	(0.95, 1.54)	2.44	(1.67, 3.58)	1.27	(0.96, 1.67)	1.29	(0.96, 1.75)	2.54	(1.74, 3.71)	1.66	(1.21, 2.27)	0.80	(0.60, 1.07)	0.93	(0.65, 1.35)
20–44	1.54	(1.20, 1.98)	3.78	(2.60, 5.51)	1.14	(0.85, 1.53)	1.39	(1.02, 1.89)	4.24	(2.93, 6.14)	1.65	(1.19, 2.28)	0.59	(0.43, 0.82)	1.21	(0.85, 1.74)
45+	2.07	(1.58, 2.73)	5.87	(3.97, 8.67)	0.89	(0.63, 1.26)	1.91	(1.38, 2.65)	7.97	(5.45, 11.65)	1.55	(1.08, 2.21)	0.44	(0.30, 0.65)	1.53	(1.04, 2.26)
*P* value trend		<0.001		<0.001		0.051		<0.001		<0.001		0.002		<0.001		0.006
*P* value trend in drinkers		0.001		<0.001		0.058		0.006		<0.001		0.267		<0.001		0.008

ORs are odds ratios of being hypertensive or in the top 20 % of the biomarker

Excludes 3 subjects in IFS2 with serum creatinine >177 mmol/L

^a^Additionally adjusted for hepatitis B and C status

^b^Ex-drinkers plus life-long abstainers combined

^c^Excludes men who drank non-beverage alcohols, as it was not possible to quantify the volume of ethanol from this source

Strong positive associations were seen for all alcohol variables in both studies with hypertension, raised HDL, ApoA1 and BNP (Table 3), for all men and also among drinkers. Of the other CVD risk factors, only triglycerides showed a systematic association with alcohol, but this was only seen for units of alcohol in the PRIME study.

With respect to BNP, the associations observed with the alcohol variables could be (1) confounded by smoking; and/or (2) mediated through the effects of alcohol on blood pressure. These possibilities are examined in Table 4, where the associations of alcohol with raised BNP levels are adjusted for these covariates sequentially. In both populations, the risk of being in the top fifth of the BNP distribution increased with level or intensity of alcohol consumption regardless of which adjustments are made. In the PRIME study, adjustment had minimal impact on strength of associations, while in IFS2 adjustment attenuated the associations slightly more. Nevertheless, even in the fully adjusted models the effects were substantial in IFS2, those with a low average intake (<5 units/week) having a considerably higher risk of raised BNP compared to non-drinkers. Most strikingly, in the fully adjusted model in IFS2, hazardous drinkers relative to non-drinkers had a four-fold increased odds of being in the top fifth of the BNP distribution.

**Table 4:** Association of BNP with measures of alcohol consumption, adjusted for various factors

		Model 1		Model 2		Model 3	
	N	OR	(95 % CI)	OR	(95 % CI)	OR	(95 % CI)
Izhevsk Family Study 2							
Alcohol biomarker quartiles^a^ (N = 958)							
1 (bottom)	246	1.00	[ref]	1.00	[ref]	1.00	[ref]
2	240	1.09	(0.64, 1.86)	1.03	(0.60, 1.76)	1.02	(0.6, 1.75)
3	237	2.07	(1.25, 3.42)	1.86	(1.11, 3.1)	1.79	(1.06, 3.01)
4 (top)	235	2.22	(1.37, 3.62)	1.94	(1.18, 3.21)	1.77	(1.05, 2.97)
*P* value trend			<0.001		0.001		0.008
Pattern of drinking (proxy report) (N = 843)							
Non-drinker^b^	120	1.00	[ref]	1.00	[ref]	1.00	[ref]
Non- hazardous	600	2.58	(1.29, 5.17)	2.75	(1.37, 5.55)	2.66	(1.32, 5.37)
Hazardous	123	4.90	(2.27, 10.6)	4.66	(2.13, 10.19)	4.16	(1.88, 9.19)
*P* value trend			<0.001		<0.001		<0.001
*P* value trend in drinkers			0.006		0.026		0.061
Alcohol units/week (self-report)^c^ (N = 915)							
Non-drinker^b^	126	1.00	[ref]	1.00	[ref]	1.00	[ref]
<5	217	1.63	(0.79, 3.35)	1.67	(0.81, 3.45)	1.67	(0.81, 3.46)
5–19	357	3.11	(1.60, 6.05)	2.99	(1.53, 5.83)	2.81	(1.43, 5.52)
20–44	151	2.84	(1.34, 5.99)	2.73	(1.29, 5.78)	2.46	(1.15, 5.26)
45+	64	3.02	(1.23, 7.39)	2.86	(1.16, 7.06)	2.58	(1.03, 6.45)
*P* value trend			0.001		0.002		0.007
*P* value trend in drinkers			0.043		0.092		0.212
Belfast PRIME study							
Alcohol units/week (self-report) (N = 1,804)							
Non-drinker^b^	399	1.00	[ref]	1.00	[ref]	1.00	[ref]
<5	419	0.98	(0.68, 1.39)	0.98	(0.69, 1.40)	0.98	(0.69, 1.41)
5–19	379	0.92	(0.64, 1.33)	0.94	(0.65, 1.37)	0.94	(0.65, 1.37)
20–44	362	1.21	(0.85, 1.74)	1.25	(0.87, 1.81)	1.23	(0.85, 1.77)
45+	245	1.53	(1.04, 2.26)	1.60	(1.08, 2.38)	1.51	(1.01, 2.25)
*P* value trend			0.006		0.004		0.012
*P* value trend in drinkers			0.008		0.003		0.012

ORs are odds ratios of being hypertensive or in the top 20 % of BNP

Excludes 3 subjects in IFS2 with serum creatinine >177 mmol/L

Model 1 − Age + obesity class (4 categories) plus waist–hip ratio (quintiles)

Model 2 − Model 1 + smoking (as never, former, current 1–20, 20+)

Model 3 − Model 2 + class of hypertension (4 categories)

^a^Adjusted for hepatitis B and C status

^b^Ex-drinkers plus lifelong abstainers combined

^c^Excludes men who drank non-beverage alcohols, as it was not possible to quantify the volume of ethanol from this source

Sensitivity analyses were undertaken (results not shown). In IFS2, adjustment for SBP and DBP as continuous variables instead of class of hypertension had no substantive effect on the findings. In addition using tobit instead of logistic regression, which allowed BNP to be treated as a continuous left-censored variable, revealed very similar effects, as did exclusion of men with angina. Analyses in PRIME excluding those with diagnosed cardiovascular disease at baseline also produced very similar results.

## Discussion

We have found that among working-age men heavy and hazardous drinking is associated with elevated levels of B-type natriuretic peptide in two population-based cross-sectional studies in Russia and the UK. This is the first time this association has been systematically investigated. These associations displayed dose–response effects overall and also when abstainers were excluded.

Alcohol and blood pressure are known to be positively associated [35], and binge drinking has been found to be associated with increased risk of stroke [16]. Blood pressure is thus an obvious potential mediator of what we have observed. Surprisingly, however, adjustment for class of hypertension (as well as SBP and DBP in IFS2), resulted in only minor attenuation of the strength of association of alcohol with BNP. Of course the information we have on blood pressure and hypertension is from one examination, and as such will not fully reflect long term differences in blood pressure. However, if blood pressure did play a major mediating role, one would expect a larger degree of attenuation.

As noted in the introduction, BNP levels may be raised as a consequence of cardiac damage caused by atherosclerotic disease. However, exclusion of men with evidence of pre-existing ischaemic heart disease or angina had little impact on the results. Unfortunately, we did not have any more direct measures of atherosclerotic disease which would have provided an even stronger base for assessing whether the association is driven by the effect of heavy drinking on coronary atherosclerosis. But in terms of lipoproteins, there was no evidence in our analyses that heavy and hazardous drinking was linked to a proatherogenic profile. There was no consistent indication that LDL and ApoB levels were elevated among heavy or hazardous drinkers, in line with the findings of a recent review of human experimental studies of the impact of alcohol on cardiovascular risk factors [36]. However, it should be noted that in the Belfast UK study, but not in Russia, there was a positive association of alcohol volume with elevated triglycerides.

The strongest associations we observed in both the Russian and the UK studies were between alcohol and HDL and Apo A1. While this is not a novel finding [36, 37], it provides further validation of the alcohol data. These effects are particularly strong in the IFS2 study in Russia, where a steep and consistent increase in risk of raised HDL and Apo A1 was seen as volume and intensity of drinking went up. Much of the literature seeking to explain the apparent protective effect of moderate alcohol consumption cites HDL as a potential underlying mechanism [38, 39]. However, there is now accumulating evidence from randomised trials and studies of genetic variants that contrary to what was inferred from observational studies [40], increasing total HDL per se may not be cardioprotective [41, 42]. Aside from this, however, it is worth noting that the mechanisms linking alcohol to HDL and Apo A1 remain elusive and poorly understood.

The Izhevsk study population had a low-risk atherogenic profile compared with the the Belfast PRIME study with lower levels of LDL and triglycerides. This parallels the findings from the Lipid Research Clinics studies in the 1980s which found similar contrasts between study sites in Russia and the USA [43]. In addition, the strength of the associations seen in Izhevsk with HDL and ApoA1 are remarkable, and are considerably steeper than in PRIME. There thus appears to be something distinctive about the impact of patterns of alcohol consumption in Russia that produce these effects that are not captured by volume of beverage ethanol, as was concluded by another study of the atypical lipid profiles found in Russia [44].

Our analyses show that, of the risk factors considered, blood pressure and BNP are the only ones that appear to be consistent with the finding from previous work that in Russia that heavy and hazardous drinking is associated with an increased risk of cardiovascular disease [22, 24]. Aside from any minor contribution via blood pressure, alcohol could also lead to an increase in BNP levels as a result of the toxic effects of ethanol or its metabolites on the myocardium, with consequent effects on cardiac contractile function, and ventricular wall stress. This may induce subclinical disease on a continuum of alcohol-induced end organ damage, at the extreme end of which is the well recognised entity of alcoholic cardiomyopathy. While the extent of cardiac contractile dysfunction may be modest, we hypothesise that this damage coupled with high peak alcohol levels (as is common in Russia) may pose a risk of life-threatening (ventricular) tachycardia and sudden death. To a lesser extent, this phenomenon may also occur among particularly heavy drinkers in PRIME and elsewhere. However, direct evidence for this is currently lacking.

The data we had and the analyses we undertook had a number of limitations. The alcohol exposure measures in PRIME were more limited than in IFS2. Moreover, even in terms of units per week, which were available in both studies, IFS2 used information relating to usual drinking over the past year, while PRIME had a reference period of the previous week. This may provide a partial explanation for the slightly weaker associations of BNP in PRIME compared to IFS2. A further lack of comparability was introduced by the fact that whereas in PRIME fasting blood samples were used, in IFS2 non-fasting samples were collected. This may partly explain the fact that there was pronounced gradient in triglycerides with increasing level of alcohol intake only in PRIME. One further limitation was the fact that we did not undertake more detailed clinical examinations in IFS2 to ascertain the cardiac status of the individuals. This would have been very valuable, and would have allowed us to look in more detail at whether the raised BNP levels associated with heavy alcohol consumption were also related to structural or functional cardiac abnormalities.

This paper also has several strengths. Firstly, having established the association of alcohol with BNP levels in the Russian study, we then replicated it in a larger study from Northern Ireland. In IFS2 we had biomarkers of alcohol consumption that showed the same pattern of associations with BNP and other outcomes as seen using the questionnaire data on alcohol. Thirdly, in both studies, BNP level was associated with breathlessness, demonstrating face validity. Finally, although BNP measurements in the two studies were carried out in different laboratories, their comparability is strengthened by the fact that they used the same type of analytic platform.

Compared to most other European countries, the pattern of alcohol consumption in Russia is particularly hazardous [45]. The Izhevsk study population reflected this, with 59 % of beverage ethanol consumed being from spirits, and 8 % of men reported to have a hangover or be excessively drunk once a week or more often. In addition, 7 % of men were reported to have drunk non-beverage alcohols in the previous year. These are relatively pure and highly concentrated sources of alcohol (typically 70–90 % ethanol by volume) that are cheap, readily available [46–48], and have been associated with particularly high mortality [23]. In contrast in PRIME only 24 % of ethanol was consumed as spirits, and there was no equivalent consumption of non-beverage alcohols.

Our findings are consistent with those of earlier studies that grappled with the paradoxical aspects of cardiovascular disease in Russia. The Lipid Research Clinics studies found that total IHD mortality in Russia showed only a very shallow decline with increasing HDL [17]. However, the risk of sudden cardiac death actually increased at higher levels of HDL. This led the authors to suggest that some of these sudden IHD deaths were misclassified alcoholic cardiomyopathies. Early evidence of this pathology has been linked to sudden death in an autopsy study in Russia [49].

If a significant proportion of the cardiac deaths attributed to ischaemic heart disease in Russia are in fact caused by alcohol-induced damage to cardiac structure and function, this has important implications for the primary prevention of cardiovascular disease at working ages. It suggests that the future development of primary care cardiovascular screening programmes will need to give particular attention to drinking behaviour. Moreover, although the wider adoption of statins and anti-hypertensives in primary prevention may have an important role to play in reducing the burden of cardiovascular disease in Russia, we speculate that this may not have as big an impact as might be anticipated on the basis of the experience in Western European populations with lower prevalences of hazardous drinking.

The data we have analysed in this paper are restricted to men from only two populations. Nevertheless, this evidence leads us to hypothesise that the relatively common patterns of hazardous alcohol drinking among Russian men may directly induce damage to the myocardium, and as a result increase the risk of cardiovascular death through impaired ventricular function and associated arrhythmias. However, at the present time evidence suitable for directly testing this is lacking, and new research aimed at exploring the cardiovascular phenotype and its correlates in heavy, spirit drinking populations such as Russia and elsewhere is now required.

## Appendix

See Table 5.

**Table 5:** Alcohol biomarkers in relation to reported alcohol consumption in IFS2 and PRIME

	Gamma-glutamyl transferase (GGT)	Carbohydrate deficient transferin (CDT)	Alcohol biomarker latent variable^a^
	Median (N)	Median (N)	Mean (N)
Izhevsk Family Study 2			
Pattern of drinking (proxy report)			
Non-drinker^b^	20.8 (130)	0.7 (128)	−0.64 (128)
Non-hazardous	31.2 (643)	1.0 (628)	0.03 (634)
Hazardous	35.1 (133)	1.7 (130)	0.41 (131)
Total	29.7 (906)	1.0 (886)	−0.01 (893)
Beverage alcohol units/week^c^ (self-report)			
Non-drinker^b^	20.0 (137)	0.6 (133)	−0.67 (135)
<5 units/week	27.2 (233)	0.8 (225)	−0.27 (231)
5–19 units/week	32.4 (373)	1.1 (366)	0.15 (367)
20–44 units/week	36.3 (162)	1.3 (160)	0.31 (158)
45+ units/week	34.4 (72)	2.3 (70)	0.50 (71)
Total	29.4 (977)	1.0 (954)	−0.01 (962)
Belfast PRIME study			
Beverage alcohol units/week (self-report)			
Non-drinker^b^	28.5 (505)	–	–
<5 units/week	33.1 (431)	–	–
5–19 units/week	38.6 (474)	–	–
20–44 units/week	41.7 (412)	–	–
45+ units/week	49.0 (291)	–	–
Total	36.9 (2,113)	–	–

^a^See statistical methods section for description of derivation of this latent variable

^b^Ex-drinkers plus life-long abstainers combined

^c^Excludes men who drank non-beverage alcohols, as it was not possible to quantify the volume of ethanol from this source
